# Bioprospecting of Marine Invertebrates for New Natural Products — A Chemical and Zoogeographical Perspective

**DOI:** 10.3390/molecules17089842

**Published:** 2012-08-16

**Authors:** Miguel Costa Leal, Carolina Madeira, Cláudio Alexandre Brandão, João Puga, Ricardo Calado

**Affiliations:** 1 Department of Biology & CESAM, University of Aveiro, Campus Universitário de Santiago, Aveiro 3810-193, Portugal; 2 Skidaway Institute of Oceanography, 10 Ocean Science Circle, Savannah, GA 31411, USA; 3 Oceanography Center, Faculty of Sciences, University of Lisbon, Campo Grande, Lisboa 1749-016, Portugal; 4 Zoo Logical, R. Brito Pais n° 8–9° E, Algés 1495-028, Portugal

**Keywords:** novel compounds, terpenoids, alkaloids, Indo-Pacific, Porifera, Cnidaria

## Abstract

Bioprospecting for new marine natural products (NPs) has increased significantly over the last decades, leading to an unprecedented discovery of new molecules. Marine invertebrates have been the most important source of these NPs, with researchers commonly targeting particular taxonomic groups, marine regions and/or molecules from specific chemical groups. The present review focuses on new NPs identified from marine invertebrates between 2000 and 2009, and performs a detailed analysis on: (1) the chemical groups of these NPs; (2) the association of particular chemical groups to specific marine invertebrate taxa; and (3) the yielding of molecules from the same chemical group from organisms occurring in a particular geographic region. Our survey revealed an increasing number of new terpenoids being discovered between 2000 and 2009, contrasting with the decreasing trend in the discovery of new alkaloids and aliphatic molecules. Overall, no particular association was identified between marine invertebrate taxa and chemical groups of new NPs. Nonetheless, it is worth noting that most NPs recorded from cnidarians and mollusks were terpenoids, while most NPs identified in echinoderms were aliphatic compounds or carbohydrates. The geographical trends observed in our study do not support the idea of particular chemical groups of new NPs being associated with marine invertebrates from any specific geographical region, as NPs from different chemical groups were commonly distributed worldwide.

## 1. Introduction

A remarkable number of new natural products (NPs) have been isolated from various marine sources in the past decades [1,2]. New NPs have provided key structures and promising compounds with the potential to be used as new therapeutic agents for a variety of diseases [3]. However, while a large number of new NPs display remarkable bioactivity and have been labeled as good candidates for potential new drugs, only a few of these NPs have successfully reached the end of the drug discovery pipeline. Nevertheless, worldwide bioprospecting efforts have not ceased, and a multitude of chemically diverse molecules have been continuously added to NP libraries [4,5,6,7,8]. Marine invertebrates are amongst the top group of organisms that have contributed with a larger number of new entries for these libraries [9,10]. These taxa have been the target of an intense scrutiny for new NPs, which have been mainly driven by unique natural features displayed by several marine invertebrates, such as the secretion of powerful chemicals to defend themselves against predation [3,11,12,13,14]. While in the past decades a large number of new NPs has been obtained from marine invertebrates, most research has only focused on less than 1% of the global biodiversity currently recognized for these taxa [15]. Under this scenario, and given the large potential of these organisms for marine drug discovery, it is likely that bioprospecting efforts will continue to target marine invertebrates in the years to come.

Drug discovery has entered a highly competitive period, where every step in the drug discovery pipeline needs to be optimized to improve the efficiency of the discovery process and maximize its outputs [4]. Regardless of the unquestionable chemical novelty of NPs discovery, their chemical diversity must start to be accessed more efficiently and effectively. As extraction and screening methods have been greatly improved in the latest years [16,17], one vital step in bioprospecting efforts that still needs further optimization is the selection of collection sites—the first step for NPs discovery. The collection site is usually selected based on a target organism(s), with high biodiversity areas commonly being the most popular [15]. Simultaneously, researchers have been narrowing their searches on particular molecules, with emphasis to the type and relevance of bioactivity displayed, in order to identify the most promising targets to include in drug discovery pipelines [18]. Particular chemical groups, such as terpenoids and alkaloids, have been unquestionably more popular among researchers searching for new NPs, as their remarkable bioactivity increase the chances for successful drug discovery and consequent patenting and commercialization [2,5,6,7,8].

The evaluation of chemical, taxonomical and geographical trends on marine NP discovery can provide important information for future bioprospecting efforts and maximize the quality and efficiency of the sampling process. In the present study we analyze the trends associated with the discovery of new NPs from marine invertebrates between 2000 and 2009 from a zoogeographical and chemical perspective. The questions addressed in this study were: (1) to which chemical groups do most new NPs from marine invertebrates discovered during the first decade of the twenty first century belong? (2) are particular chemical groups associated with specific marine invertebrate taxa? and (3) have molecules from the same chemical groups been isolated from organisms occurring in a particular geographic region?

## 2. Results

### 2.1. Chemical Trends

**Figure 1:**
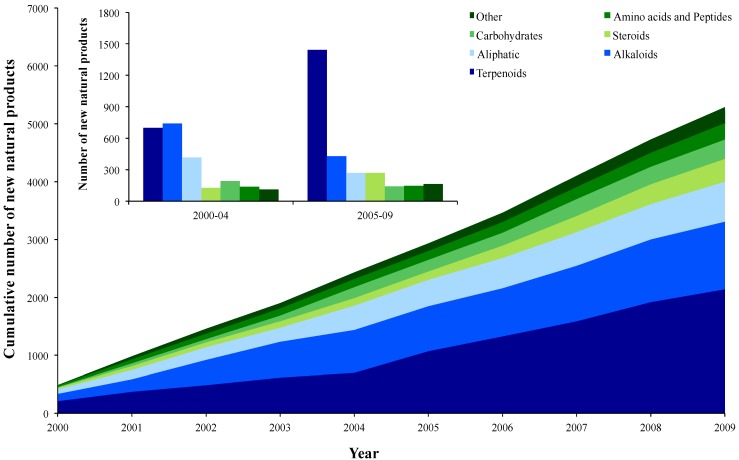
Chemical groups of new marine natural products from invertebrates. Cumulative number of new natural products from different chemical groups discovered from 2000 to 2009 (group “Other” include polyketides, simple aromatic, polypyrroles and oxygen heterocycles). Inset: Total number of new marine natural products from invertebrates discovered between 2000–2004 and 2005–2009 according to chemical group.

The present work covered a total of 5,286 NPs discovered from 2000 to 2009. Most NPs recorded were terpenoids (40.5%). Alkaloids (22.1%), aliphatic compounds (13.0%), steroids (7.5%), carbohydrates (6.3%) and amino acids and peptides (5.4%) also accounted for a notable number of NPs. During the first decade of the twenty first century an increasing trend in the discovery of new terpenoids has been recorded, with alkaloids exhibiting an opposite trend (Figure 1). The yearly average number of new terpenoids discovered between 2005–2009 (288.4 ± 61.4; average ± standard deviation) was twice the number recorded between 2000–2004 (140.0 ± 49.3). Contrasting results were observed for alkaloids, as the number of new NPs decreased between 2000 and 2009 from 148.4 ± 53.2 NP year^−1^ to 85.6 ± 41.8 NP year^−1^, respectively.

### 2.2. Taxonomical Trends

Between 2000 and 2009, most NPs from marine invertebrates were isolated from the phyla Porifera (47.1%) and Cnidaria (33.5%). Although in comparably lower numbers, the phyla Echinodermata (7.4%), Chordata (6.0%), and Mollusca (5.0%) also accounted for a large number of new NPs. This trend did not hold up when the finding of NPs was analyzed by chemical group, as the number of new terpenoids, aliphatic compounds, or any other particular group, was not always dominated by organisms from the phyla Porifera and Cnidaria (Table 1).

**Table 1:** Percentage (%) of new marine natural products from invertebrate sources considering the different chemical groups and corresponding phyla.

Phyla	Terpenoids	Alkaloids	Aliphatic	Steroids	Carbohydrates	Amino acids and
	(%)	(%)	(%)	(%)	(%)	peptides (%)
Porifera	30.0	30.9	12.0	6.5	4.9	8.1
Cnidaria	65.9	10.1	7.7	8.9	6.1	0.4
Echinodermata	10.5	10.7	35.8	14.3	20.7	0.8
Chordata	23.0	39.4	16.1	2.8	4.4	7.9
Mollusca	42.6	12.5	19.0	1.1	3.0	17.1

Note: Only 99% of the phyla providing new natural products discovered between 2000 and 2009 and chemical groups representing 95% of new natural products are displayed.

Table 1 shows that a notable fraction of NPs discovered from cnidarians and mollusks were terpenoids, whereas alkaloids were dominant among new NPs from the phylum Chordata (tunicates; see Experimental section). NPs recovered from sponges (phylum Porifera) were mostly alkaloids and terpenoids, while the largest number of new NPs from echinoderms were aliphatic compounds. It is also important to note which phyla showed the highest percentage of a particular chemical group. Of all terpenoids covered in this study (2,142 NPs), 54.5% were isolated from the phylum Cnidaria. Alkaloids, aliphatic compounds, and amino acids and peptides were mostly obtained from Porifera (65.6% of 1170 NPs, 43.6% of 686 NPs, and 70.8% of 284 NPs, respectively). Both steroids (total of 398 NPs) and carbohydrates (total of 332 NPs) were relatively abundant among the phyla Porifera (40.7% of steroids and 36.5% of carbohydrates) and Cnidaria (39.5% of steroids and 32.5% of carbohydrates).

### 2.3. Zoogeographical Trends

Most NPs covered in this study were obtained from invertebrates sampled in marine regions bordering Asian marine areas (55.1%). In decreasing order of importance, all other NPs were recovered from countries located in Oceania (16.5%), America (14.6%), Africa (6.1%) and Europe (4.5%). The analysis of each chemical group in separate, *i.e.*, considering the total number of NPs discovered per chemical group as 100%, revealed a similar trend among different continents. In other words, for each chemical group, new NPs from Asian marine areas accounted for 42%–67% (minimum-maximum), followed by NPs from waters of Oceania 7.8%–33.8%, America 11.3%–25.6%, Africa 2.8%–7.0% and Europe 3.3%–6.0%. However, when analyzing the different chemical groups for each geographical zone (considering the total number of NPs discovered per geographical zone as 100%), the observed trend varies from the overall trend for the chemical groups (see Section 2.1). Terpenoids accounted for 40.7% and 45.9% of new NPs discovered in Africa and Asia, respectively. NPs from American marine areas were mostly terpenoids (32.9%) and alkaloids (26.6%), as well as NPs from European countries (30.1% of terpenoids and 28.8% of alkaloids). In contrast, NPs from Oceania are mostly aliphatic compounds (31.5% of new NPs), whereas terpenoids accounted for 22.2% of the new NPs. Most NPs discovered between 2000 and 2009 were yielded by invertebrates from tropical regions from the Southern Hemisphere (Table 2). The most noticeable increase in the discovery of NPs during the study period was recorded for terpenoids in tropical regions, whereas an overall decrease of alkaloids and aliphatic compounds was observed for all regions.

**Table 2:** Number of new marine natural products from invertebrates discovered during the first (2000–2004) and second half (2005–2009) of the first decade of the twenty first century according to chemical groups and latitudinal regions where target organisms were sampled.

Hemisphere	Region	Terpenoids		Alkaloids		Aliphatic		Steroids		Carbohydrates		Amino acids and peptides	
		2000–2004	2005–2009	2000–2004	2005–2009	2000–2004	2005–2009	2000–2004	2005–2009	2000–2004	2005–2009	2000–2004	2005–2009
North	Polar	0	0	2	3	4	0	0	0	0	0	1	0
	Temperate	222	281	251	121	179	136	55	52	55	56	18	42
	Tropical	176	395	200	111	38	63	32	86	55	67	53	41
South	Tropical	202	643	216	147	111	65	35	94	49	10	42	50
	Temperate	36	42	48	31	35	1	4	14	5	2	10	6
	Polar	25	24	5	8	10	0	0	10	7	5	2	0

Note: Only the 95% of the new NPs from all the chemical groups discovered between 2000 and 2009 are represented.

**Figure 2:**
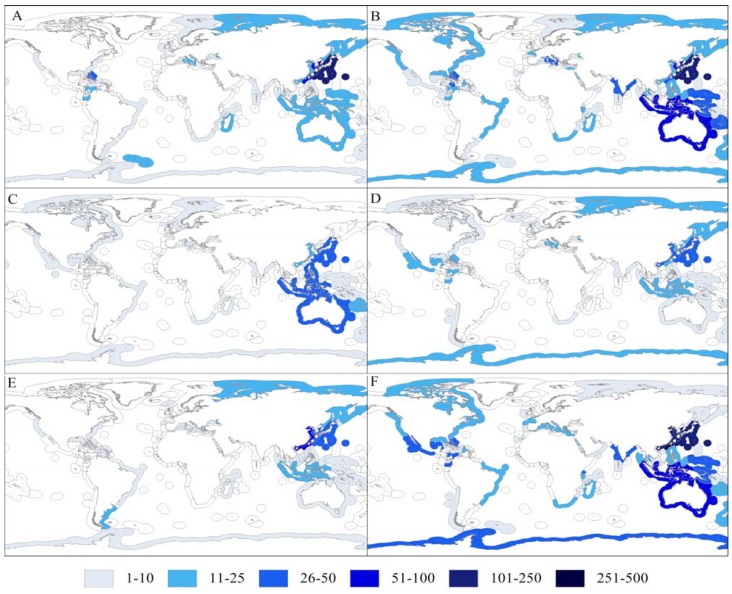
Exclusive Economic Zones. Number of new natural products discovered from marine invertebrates for world Economic Exclusive Zones (EEZ) between 2000 and 2009 according with chemical groups: (**A**) terpenoids; (**B**) alkaloids; (**C**) aliphatic; (**D**) steroids; (**E**) carbohydrates; (**F**) amino acids and peptides.

The distribution of new NPs from invertebrates according to exclusive economic zones (EEZs) and chemical group is illustrated in Figure 2. Most terpenoids were associated with the Taiwanese EEZ (23.2% of all terpenoids), as well as Chinese (12.0%) and Japanese (11.7%) EEZs. All other EEZs accounted for less than 6% of all terpenoids. The Japanese EEZ was also an important geographic area for the discovery of new alkaloids (16.0%) and aliphatic compounds (22.7%). A relatively large percentage of new aliphatic NPs was also recovered from South Korean and Taiwanese EEZs (15.3 and 8.8%, respectively). The Taiwanese EEZ was also associated with the largest fraction of steroids discovered during the study period (20.9%). The overall trends observed in Figure 2 show that Indo-Pacific countries have been a notable source of NPs from various chemical groups. Besides EEZs located in the Indo-Pacific, those from the Caribbean have also yielded a relatively high number of new terpenoids and alkaloids (Figure 2A,B). In contrast, only the EEZs from the Indo-Pacific (and Russia EEZ in particular for steroids) have shown a relatively higher number of new amino acids and peptides and steroids (Figure 2D,F, respectively). Although with relatively lower numbers when compared with Indo-Pacific EEZs, the Antarctic EEZ also showed a notable number of terpenoids and carbohydrates (Figure 2B,E).

Table 3 shows the percentage of new NPs from different chemical groups taking into consideration the total number of NPs discovered in each EEZ. Most EEZs show the trend already described: relatively higher numbers of terpenoids, followed by alkaloids and aliphatic compounds (see Sections 2.1 and Sections 2.2). The EEZs of China, Taiwan and Mexico should be highlighted by the pronounced contribution of terpenoids for the total number of NPs discovered in these regions (at least 60% of all new NPs from marine invertebrates) (Table 2). In contrast, a larger percentage of alkaloids was associated with the EEZs of Fiji, Italy, Madagascar, New Zealand, Palau and Vanuatu. Philippine and Russian EEZs should also be noted, as they exhibited a relatively large fraction of amino acids and peptides and carbohydrates, respectively.

**Table 3:** Percentage of new marine natural products (NPs) from invertebrates from different chemical groups reported from different Economic Exclusive Zones (EEZs). The total number of new NPs from marine invertebrates discovered in each EEZ is also presented.

Exclusive Economic Zone	Terpenoids (%)	Alkaloids (%)	Aliphatic (%)	Steroids (%)	Carbohydrates (%)	Amino acids and peptides (%)	Total
Antarctica	42.5	13.8	12.5	12.5	15.0	2.5	80
Australia	37.5	33.5	6.0	2.8	3.6	10.9	248
Bahamas	22.4	28.0	28.0	3.2	10.4	1.6	125
China	63.3	3.8	3.3	14.9	8.7	3.3	390
Colombia	46.0	18.4	19.5	0.0	16.1	0.0	87
Fiji	16.7	36.7	23.3	0.0	0.0	16.7	60
India	47.7	34.9	3.5	0.0	7.0	3.5	86
Indonesia	33.9	31.8	6.4	6.4	4.2	10.2	283
Italy	17.5	33.8	16.3	5.0	13.8	3.8	80
Japan	33.6	25.7	20.5	3.6	4.7	5.4	717
Madagascar	21.1	36.8	21.1	5.3	0.0	7.0	57
Mexico	59.5	3.8	0.0	0.0	30.4	6.3	79
Micronesia	32.4	29.5	18.1	3.8	4.8	7.6	105
New Zealand	29.7	37.5	12.5	0.0	3.1	6.3	64
Palau	21.3	31.3	0.0	10.0	3.8	10.0	80
Papua New Guinea	39.5	31.5	12.9	1.6	3.2	6.5	124
Philippines	24.2	18.2	4.0	9.1	7.1	26.3	99
Russia	7.1	14.3	21.4	20.2	26.2	0.0	84
South Korea	34.9	17.4	28.3	11.1	6.9	0.0	350
Taiwan	69.6	6.0	8.3	11.7	2.6	0.1	684
United States	33.3	30.2	15.9	9.5	1.6	1.6	63
Vanuatu	18.6	48.8	4.7	8.1	1.2	12.8	86

Note: Only EEZs from which more than 1% of all new NPs from invertebrate sources discovered between 2000 and 2009 are represented. Only the chemical groups of the 95% of new NPs are shown.

## 3. Discussion

This study investigated the chemical, taxonomical and geographical trends of NPs discovery from 2000 to 2009 of marine invertebrates. If a particular group (taxonomic or geographic) does not show a new NP, it means that no NP was isolated from that group in the past decade. It does not necessarily mean that a given taxonomic group does not synthesize a NP that is already known. The present study does not aim to provide a comprehensive overview of all NPs from marine invertebrates (for that purpose please refer to marine chemical ecology reviews already published, such as [19,20,21]), nor does it attempt to provide unconditional statements about the discovery of NPs.

### 3.1. Chemical Trends

The discovery process of new NPs starts with sampling and is followed by several steps, such as sample preservation, extraction protocol and further laboratory processing [3]. The selection of the target organism to be screened, along with the laboratory procedures that will ultimately lead to the discovery of NPs, is usually decided by researchers. The rationale for these choices is commonly based on the taxonomic relevance of the biological sample or the chemical profile of the particular group of molecules being targeted. Marine invertebrates synthesize primary and secondary metabolites that are ultimately screened and described by researchers as NPs. Primary metabolites, which include amino acids, simple sugars, nucleic acids and lipids, are molecules necessary for cellular processes and essential for an organism to survive. In contrast, secondary metabolites, such as alkaloids and terpenoids, are not directly involved in critical physiological processes, and often play a role in interspecific and other ecological interactions. In terrestrial ecosystems, particularly in plants, terpenoids are known to be very abundant and structurally diverse [22]. Terpenoids are also the chemical group that includes most NPs isolated so far from marine environments [2,23], as also confirmed in the present study for marine invertebrates. Terpenoids display an immense variety of structural types, which is in part associated with the fact that their biosynthetic unit can be rearranged and highly oxidized [24,25]. Terpenoids also display a wide array of known bioactivities and biological functions [11,12,13,14,18]. These features support their use in the pharmaceutical and food industry for their potential and effectiveness as medicines and flavor enhancers, respectively [5,6,22]. The high biotechnological potential of terpenoids helps to explain the trends recorded in the present study, namely the increasing number of new terpenoids discovered between 2000 and 2009 in comparison to alkaloids and aliphatic NPs (Figure 1). Alkaloids are also known to be biogenetically and structurally diverse, with its terminology being historically associated with pharmacologically active basis [25]. It may be legitimate to assume that the decreasing trend recorded in new records of alkaloids may reflect the increasing interest of researchers on terpenoids and, consequently, a shift in the main chemical group being targeted in bioprospecting efforts between 2000 and 2009.

### 3.2. Taxonomical Trends

As already noted on several studies [2,10,15], Porifera and Cnidaria are the marine phyla with the largest number of new NPs discovered between 2000 and 2009. This does not necessarily mean that sponges and cnidarians have a larger diversity of NPs than other marine invertebrate groups. This trend probably results from the popularity of these organisms among researchers performing bioprospecting studies and the positive discrimination towards sponges and cnidarians over other marine invertebrates [26]. Nevertheless, the observed trends for NPs discovered in different chemical groups within each phyla (Table 1) suggests one of the following hypothesis: (1) researchers looking for new NPs favor particular chemical groups when targeting particular phyla; (2) organisms from different phyla display higher diversity of NPs of particular chemical groups. For instance, most new NPs recorded from echinoderms were aliphatic and carbohydrates. This result indicates that either researchers targeting echinoderms were more frequently looking for aliphatic or carbohydrates NPs or that echinoderms have indeed a higher diversity of aliphatic and carbohydrates in comparison to NPs of other chemical groups. In contrast, as organisms from the phylum Porifera are among the most targeted marine organisms by chemical biologists [10], results observed for this group reflect the bioprospecting popularity of terpenoids and alkaloids among researchers. Such hypothesis is reinforced with the dominance of Porifera’ NPs recorded in our study when accounting for each chemical group in separate. The phylum Cnidaria, for instance, displayed a notable higher fraction of terpenoids in comparison with Porifera. Such result may be associated with the increasing popularity of cnidarians in bioprospecting efforts in the twenty first century [10,15], together with the growing popularity of terpenoids among researchers searching for new NPs. The relatively high percentage of amino acids and peptides isolated from the phylum Mollusca must also be emphasized, as it is generally known that mollusks, particularly sea slugs and sea snails, possess very potent venoms (commonly peptide-based molecules) [16,27]. It is possible that the trends recorded in our study are simply associated with an intentional bias of researchers investigating these mollusks towards the analysis of amino acids and peptides. Nevertheless, this does not necessarily mean that mollusks produce more metabolites from *de novo* synthesis, as some of their NPs may originate from dietary sources (e.g., corals, bryozoans, sponges…). Furthermore, while the taxonomical analysis of our results may allow the speculation of particular chemical groups of NPs being more common in certain taxa, it is important to note that some of the metabolites obtained from invertebrates may be synthetized by symbiotic microbes. This issue is particularly relevant in groups such as Porifera, Cnidaria and Chordata, as these taxa are already known to be rich in symbiotic microbes that may provide their hosts with primary and secondary metabolites [28,29,30]. 

### 3.3. Geographical Trends

The notable numbers of new NPs from marine invertebrates recovered from Indo-Pacific organisms has already been highlighted in a previous study [15]. Furthermore, particular emphasis should be given to Chinese and Taiwanese EEZs, as a high number of terpenoids was observed when compared to other chemical groups. Such trends might be associated with the increasing bioprospecting efforts registered in these countries after the year 2000, along with the increasing scientific and economic interest in terpenoids [22]. However, if one considers the hypothesis that the trends recorded suggest researchers’ preferences rather than a differential chemical diversity in specific regions, it is difficult to interpret the dominance of aliphatic NPs from Oceania and the increasing trend of new sterols being discovered in the southern pole, *i.e.*, Antarctica EEZ (Figure 2C, Table 2). The popularity of tropical regions over other habitats for bioprospecting is further reinforced, probably because they harbor most marine biodiversity hotspots and/or logistics and costs associated with bioprospecting expeditions in these regions are more appealing to researchers. This may justify why relatively fewer NPs discoveries have been made in polar areas, particularly Antarctica. Although Antarctic waters show high biodiversity, its inaccessibility is the most likely cause for the low number of new NPs recorded so far for this region [11].

## 4. Experimental

### Methods

The reviews of Marine Natural Products published every year by Natural Product Reports were examined in order to gather available information on new NPs from marine invertebrates. Information for the years 2000 to 2009 was assembled [1,10,31,32,33,34,35,36,37,38]. Information on source organisms, particularly taxonomical information and collection sites, was gathered along with the NP discovered and its chemical group. When particular information was insufficient or omitted, the original article describing the discovery of the NP was accessed, in order to retrieve data as accurately as possible. Note that it was not always possible to retrieve all missing information by consulting the original article, as some of those works were written in languages other than English or no detailed information was provided on the geographical location of sampling site. About 8% of all NPs recorded in the present study lacked sufficient information for one of these criteria.

The World Register of Marine Species (WoRMS) database was used to provide detailed taxonomical information for each species and to validate and/or update their scientific names [39]. WoRMS database was also used to determine the total number of species currently recognized as taxonomically valid that belonged to distinctive marine invertebrate phyla. Although several studies addressing NP from marine invertebrates commonly include tunicates, this group of organisms belongs to phylum Chordata [15,39,40]. In this way, while NPs isolated from tunicates were also considered in the present work, every time that Chordata is mentioned throughout the text it refers exclusively to tunicates.

Information on the collection site of each source organism was used to identify each NP. This information was used to determine the following geographical categories: continent, latitude and EEZ (list available at www.seaaroundus.org/eez/). Six continents (Africa, America, Antarctica, Asia, Europe, Oceania) were defined. Latitude was organized in polar (above the Arctic Circle and below the Antarctic Circle), temperate (between the Tropic of Cancer and the Arctic Circle and between the Tropic of Capricorn and the Antarctic Circle) and tropical (between the Tropic of Cancer and Tropic of Capricorn), and each was further divided in North and South. Concerning EEZ, data of external territories, such as provinces, overseas departments, *etc*., were separated from their parent country. In the present study, the information concerning those external territories was treated as a separate EEZ. The geographical information was mapped using Manifold® 8.0 software.

All NP information was also grouped in chemical categories as defined in the Dictionary of Marine Natural Products [25]. Accordingly, the following groups were selected: aliphatic, carbohydrates, oxygen heterocycles, simple aromatic, terpenoids, steroids, amino acids and peptides, alkaloids, and polypyrroles. As the present study analyzed all NPs from marine invertebrates discovered from 2000 to 2009, no statistical analyses were conducted to determine surveyed trends over this period.

## 5. Conclusions

The aim of this work was to identify potential chemical, taxonomical and geographical trends on bioprospecting efforts for new NPs from marine invertebrates between 2000 and 2009. Our results clearly evidence an increasing interest towards new terpenoids, while the discovery of new alkaloids and aliphatic NPs has decreased over the same period. In general, there was no particular chemical groups obtained from specific taxa, even though a notable fraction of new NPs from phylum Cnidaria and Mollusca were terpenoids and the majority of new NPs from phylum Echinodermata were either aliphatic or carbohydrates. NPs from the same chemical group have been yielded from organisms occurring worldwide and were not exclusively present in any given geographic region. If the growing suspicion that many NPs obtained from marine invertebrates have their real origin from associated microbes, the taxonomical and zoogeographical trends observed in this study may be associated with the symbiotic community present in the analyzed invertebrates.

The systematic analysis of overall trends exhibited in bioprospecting may allow researchers to redirect their efforts towards different taxonomical groups or geographic regions, in order to improve the efficiency of their studies and maximize the number of new NPs being discovered. A distinct approach that may be followed in future bioprospecting studies by more conservative researchers is to simply focus their bioprospecting efforts on taxonomic groups or geographical regions that have already yielded significant numbers of new NPs. While marine biodiversity is already acknowledged as a major resource for human societies, the assessment of its true value as a source of new compounds with biotechnological applications (with emphasis to new pharmaceuticals) still requires further study. It will only be possible to merge bioprospecting interests and conservation efforts, along with social, ecological and financial sustainability, by fully recognizing the importance of marine biodiversity for industrial applications of any type.
